# Editorial: Rising stars in precise sustainable irrigation

**DOI:** 10.3389/fpls.2023.1257658

**Published:** 2023-08-02

**Authors:** María R. Conesa, José F. Maestre-Valero, Victor Blanco

**Affiliations:** ^1^ Department of Irrigation, Centro de Edafología y Biología Aplicada del Segura – Consejo Superior de Investigaciones Científicas (CEBAS-CSIC), Murcia, Spain; ^2^ Department of Agricultural Engineering, Universidad Politécnica de Cartagena (UPCT), Cartagena, Spain; ^3^ Department of Horticulture, Washington State University, Pullman, WA, United States

**Keywords:** deficit irrigation, irrigation water use efficiency (IWUE), fertigation and nutrient content, remote sensing, soil and plant water relations

Irrigation is a fundamental requirement for agricultural production in arid and semi-arid areas subjected to water scarcity. Sustainable crop productivity depends on precise and effective irrigation regimes. Furthermore, inadequate irrigation management can result in plant water stress due to the deficit or excess of water, penalizing crop yield and quality. To overcome the challenge of feeding the ever-increasing world population and the limited availability of water exacerbated by climate change, Irrigation Water Use Efficiency (IWUE) must be increased.

Precise sustainable irrigation (PSI), which involves rational irrigation strategies (such as deficit irrigation and/or use of non-conventional water resources for irrigation) or systems based on digital technologies (automation and remote sensing), have proven to be effective tools for increasing IWUE (Vories and Sudduth, 2021).

This Research Topic contains five original articles from recognized early career researchers (Rising Stars researchers). It gathers novel insights and innovative knowledge, technology and agricultural practices related to PSI.

Soil and plant-based water status indicators are often proposed as key inputs for irrigation management in decision support systems (DSS). Precision irrigation technologies using sensors feedback can provide automated DSS for irrigation scheduling and help farmers to implement more efficient deficit irrigation strategies (Osroosh et al., 2016; Vera et al., 2021).

One of the most used sensor-based irrigation scheduling system is the Irrigation Scheduling Supervisory Control and Data Acquisition (ISSCADA) System patented by Evett et al. (2014). A two-year experiment in cotton by O’ Shaughnessy et al. compared the irrigation applied and crop response between automated irrigation managed with ISSCADA at different levels of soil water depletion, and manual irrigation scheduling using weekly neutron probe readings at full-irrigation conditions. The automated irrigation methods were based on ISSCADA-plant feedback (crop water stress index (CWSI) threshold values) and ISSCADA-hybrid irrigation scheduling method (combining CWSI and volumetric soil water content (θ_v_) threshold values). The findings showed that the agronomical response of automated ISSCADA irrigation methods performed similarly to manual irrigation scheduling methods, but the last one was higher time-consuming and required labour intensive use along with qualified personal staff to measure with neutron probes (Vera et al., 2017). Comparing both ISSCADA methods, ISSCADA-plant feedback resulted in significantly less water applied but remains constant productive parameters. However, the authors noted the benefit of including a second sensor feedback method for the security of proper irrigation scheduling.

In the work of Conesa et al. the automated irrigation method was based on real-time θ_v_ values computed using the management allowed depletion (MAD) concept (Merriam, 1966). The MAD-based irrigation protocol was validated with novel trunk microtensiometers (MTs; FloraPulse, Davis, CA, USA). These biosensors are directly embedded into the tree woody tissue and provide continuous information about trunk water potential (Ψ_trunk_) using a microelectromechanical pressure sensor. Ψ_trunk_ measurements involve the complete water pathway from the roots, which absorb the water available in the soil, to the stem whereby water is transported through the xylem, to fruit and leaves where it evaporates into the air (transpiration *via* stomata). Moreover, MTs have the advantage of providing real-time data in easy-to-interpret pressure units (Blanco and Kalcsits, 2021). The results obtained showed that measurements of Ψ_trunk_ were able to assess plant water status in nectarine trees. Furthermore, they can be used as a promising alternative to discrete measurements of leaf (Ψ_leaf_) or stem water potentials (Ψ_stem_) measured with traditional pressure chamber, which cannot be automated.

Thermal-based indicators can be an alternative for assessing plant water status in a rapid, non-destructive and cost-effective way (Blaya-Ros et al., 2020). Canopy temperature (Tc) is a plant water status indicator based on the leaf energy balance. When plants are under water stress, they respond with a partial stomatal closure, reducing the stomatal conductance (g_s_), limiting leaf transpiration and promoting an attenuation of the evaporative cooling process, resulting in higher Tc values (Jones, 2004). However, Tc does not only depend on the stomatal aperture but also on other agrometeorological variables such as solar incidence angle, solar radiation, air temperature (Ta) and wind speed. For this reason, the canopy-to-air temperature difference (Tc - Ta; Idso et al., 1981), and the crop water stress index (CWSI; Jackson et al., 1981) have been developed to minimize the effects of environmental variables on the absolute Tc values. In the work of Blanco et al., Tc-Ta and CWSI were strongly related to Ψ_stem_ and g_s_, however, they were not able to detect water deficit earlier than θ_v_ sensors, which did not result as related to the tree water status as the thermal-based indices. The work proposes a multiple regression analysis that combines thermal base-indices and θ_v_ sensors which provides accurate information on tree water status and overcomes the limitations of the individual use of each indicator.

Hyperspectral techniques were analysed with different variable selection methods in the study of Shi et al. to predict soil salinity which is another main cause of land degradation in arid and semi-arid areas. Moreover, high soil salinity affects water absorption by plants, inhibiting plant dry matter accumulation (Ahmad et al., 2002). Hyperspectral remote sensing data provide complete information of crop spectrum which is useful to monitor soil salinity. However, it also requires of specific analysis to reduce the redundancy and complexity. The authors proposed a method for predicting accuracy of root zone soil salinity under vegetation cover conditions by combining vegetation canopy spectral and crop aboveground growth parameters.

Finally, Imbernon-Mulero et al. presented and validated an autonomous system integrated into the irrigation head for optimal management of fertigation called NutriBalance. It was designed to manage irrigation waters of different qualities by considering different prices scenarios, fertilizers and crop nutrient requirements. The results showed good interoperability between NutriBalance and the irrigation head which was able to prescribe optimal fertigation under different scenarios.

## Author contributions

MC: Conceptualization, Funding acquisition, Investigation, Validation, Writing – original draft, Supervision. JM-V: Conceptualization, Funding acquisition, Validation, Writing –review & editing, Investigation, Project administration, Supervision. VB: Conceptualization, Funding acquisition, Investigation, Supervision, Validation, Writing – review & editing. All authors listed have made a substantial, direct and intellectual contribution to the work and approved it for publication.
